# Supplementary material for: Synthesis runs counter to directional folding of a nascent protein domain
Source: Nat Commun. 2020 Oct 9;11:5096. doi: 10.1038/s41467-020-18921-8 (PMC7547688; doi:10.1038/s41467-020-18921-8)
Supplement: Supplementary file 1 — Supplementary Information [file 41467_2020_18921_MOESM1_ESM.pdf]

Supplementary Information

Synthesis runs counter to directional folding of a nascent protein domain

Xiuqi Chen [1], Nandakumar Rajasekaran [1], Kaixian Liu [1,‡] and Christian M. Kaiser [2,3,*]

[1] CMDB Graduate Program, Johns Hopkins University, Baltimore, MD, USA

[2] Department of Biology, Johns Hopkins University, Baltimore, MD, USA

[3] Department of Biophysics, Johns Hopkins University, Baltimore, MD, USA

[‡] present address: Molecular Biology Program, Sloan Kettering Institute, New York, NY

[*] corresponding author (kaiser@jhu.edu)

Supplementary Information includes

- Supplementary Figures 1 to 7
- Supplementary Tables 1 to 3
- Supplementary References

Supplementary Figures

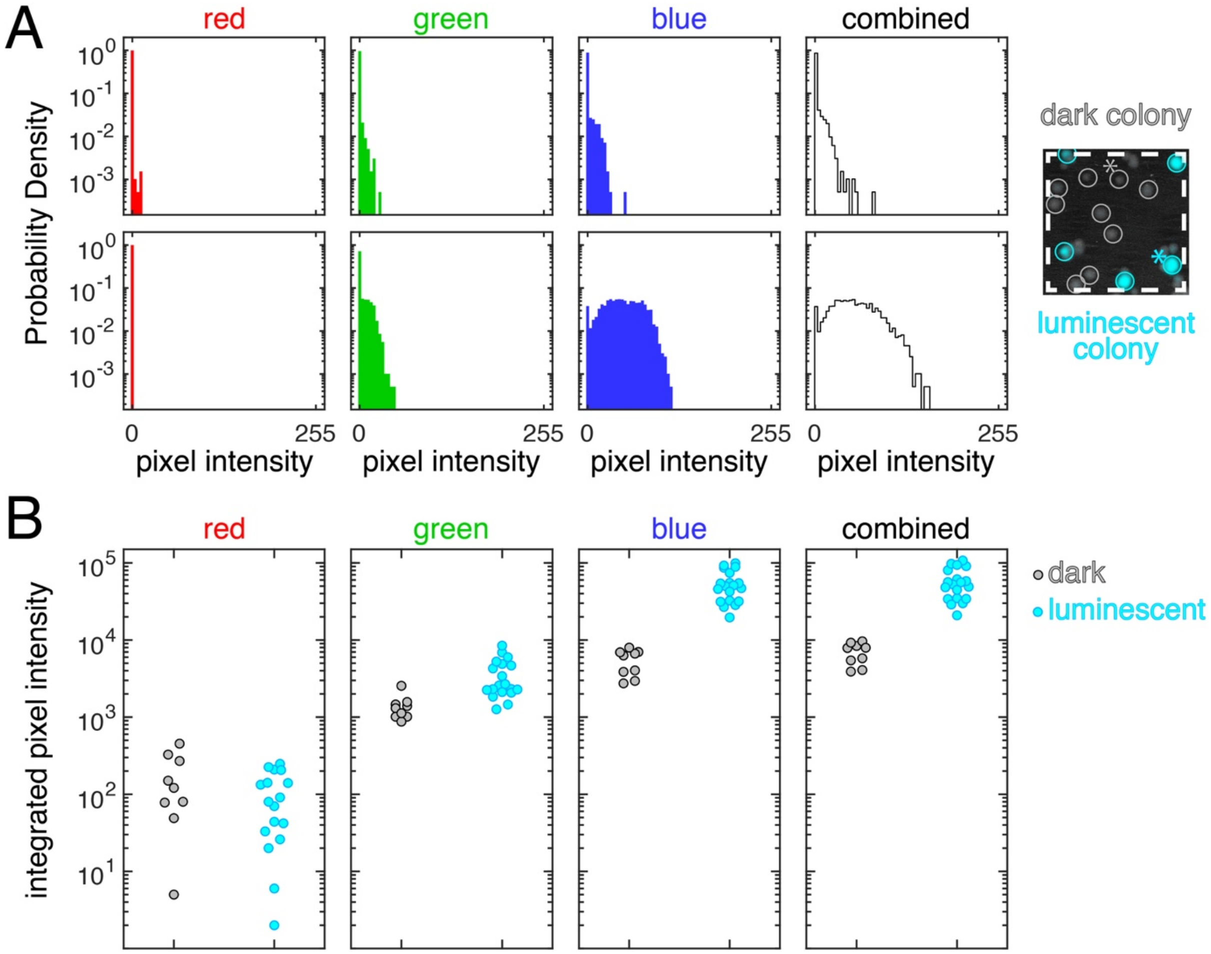

Supplementary Figure 1: Quantification of colony luminescence. A. Histograms of pixel intensities (8-bit rgb image) for the individual channels (red, green, blue) and for the combined channels (combined) for a weakly luminescent colony (top) and a highly luminescent colony (bottom). The colonies for which the example histograms are shown are marked with asterisks in the panel on the right (same as in Figure 3C). Most of the luminescence signal is detected in the blue channel, consistent with the NanoLuc emission maximum [1] at a wavelength of 460 nm. **B.** Univariate scatter plots of the integrated pixel intensity (sum of all pixel values) for the sequenced colonies shown in Figure 3D. Luminescent colonies are clearly separated from non-luminescent colonies in the blue channel and in the combined channels. The lowest value in the luminescent colony group is more than twice as large as the highest value in the dark colony group. For all plots, the y-axis is formatted on a logarithmic scale to visualize the widely differing values for the individual channels.

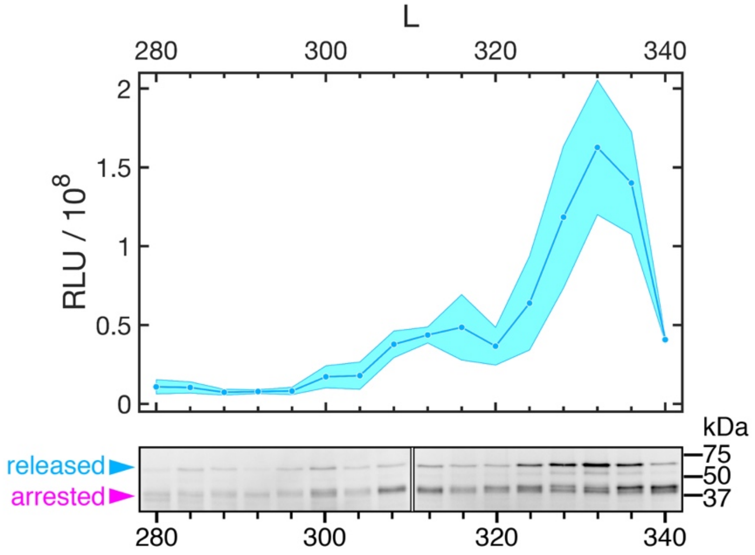

Supplementary Figure 2. Visualization of translation products by Western blotting. The top panel shows the luminescence in the region 280 ≤ L ≤ 340 (data from Figure 4; see figure caption for details; the blue line represents the mean from 3 independent replicates, the cyan colored regions represents the standard deviation). The bottom panel shows Western blots generated from samples in this length range, with the approximate positions of arrested and released products indicated by magenta and blue arrowheads. The band intensities for the released products reflect the measured luciferase activities, indicating that the low luminescence reading in the region of L ≤ 300 is not due to the accumulation of released product in which the reporter is inactive as a result of being fused to long polypeptides lacking stable structure. Luciferase activity therefore appears to faithfully report on release of SecM-mediated arrest.

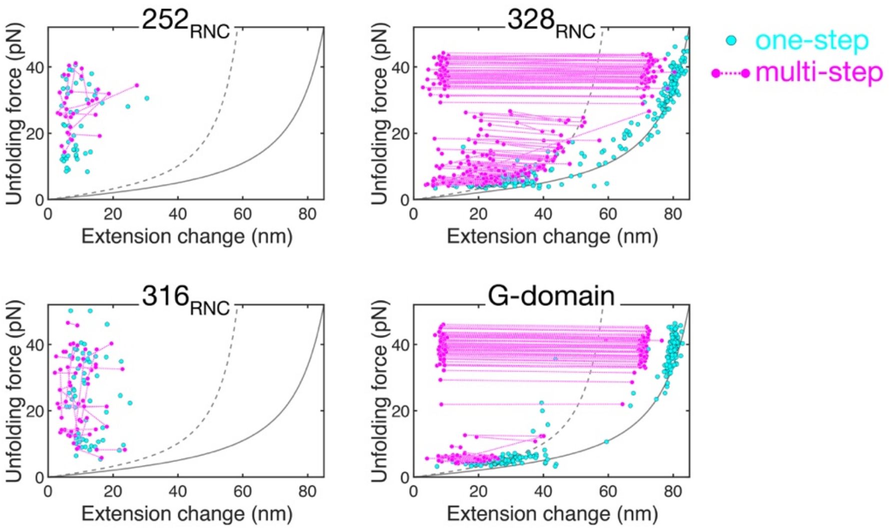

Supplementary Figure 3. Unfolding transitions from force ramp experiments. The scatter plots show the transitions observed in force-ramp measurements of nascent EF-G polypeptides (252_{RNC}, 17 molecules; 316_{RNC}, 48 molecules; 328_{RNC}, 30 molecules) and the isolated G-domain (16 molecules). Every circle represents an unfolding event, characterized by the force at which it occurs and the associated change in molecular extension (see Liu *et al*. [2] for a detailed description). Single unfolding events are shown in cyan. Serial transitions from the same force-extension curve are represented as magenta circles, connected by dotted lines. 328_{RNC} and the G-domain show defined unfolding transitions in the range between ~30 pN to ~45 pN, characteristic of G-domain unfolding [2,3]. 328_{RNC} also shows transitions resulting from the incompletely folded G-domain. In contrast, transitions recorded with 252_{RNC} and 316_{RNC} are short, heterogeneous and spread over a wide force range. Almost all 328_{RNC} and G-domain traces contained measurable transitions. Transitions were observed infrequently for the shorter nascent chains (252_{RNC}: 60 traces with transitions, 213 traces total; 316_{RNC}: 85 traces with transitions, 363 traces total). WLC-models with contour lengths of L_C = 103.7 nm (full G-domain unfolding) and L_C = 73.1 nm (intermediate lacking the C-terminal 90 amino acids; see main text for details) are indicated by solid and dashed grey lines, respectively. The data shown for 328_{RNC} and the G-domain have previously been published [3].

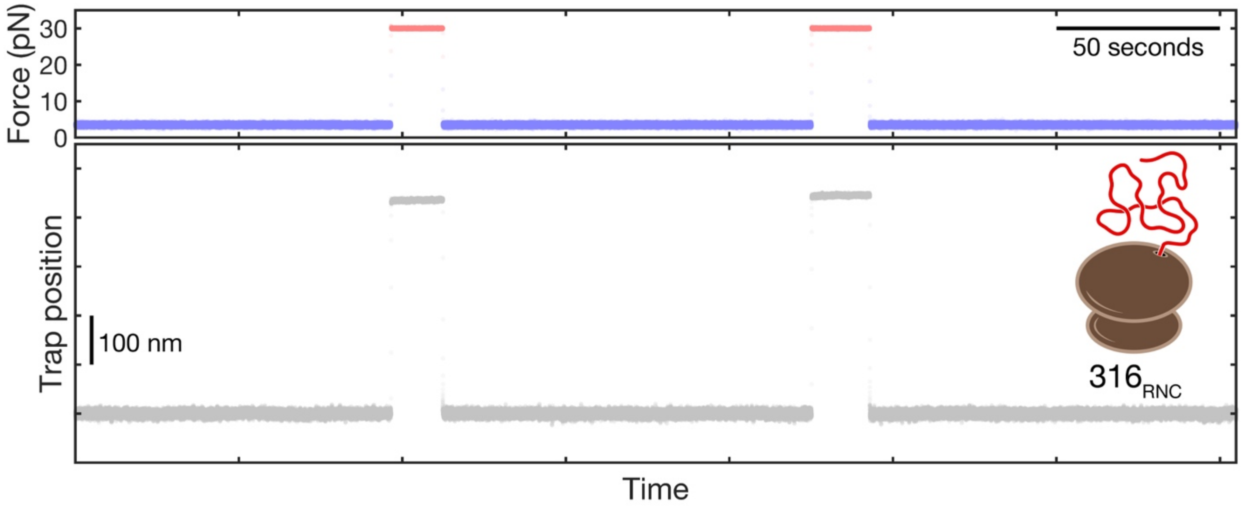

Supplementary Figure 4. Constant force recording of 316_{RNC}. The top panel shows the measured force, which is jumped between 3.5 and 30 pN. The bottom panel shows the measured raw data, trap position, which includes the extension of the nascent chain, as well as the extension of the DNA handles and the bead displacement from the trap center. The latter two contributions (from DNA handles and bead displacement) are constant at a given force, and changes in trap position reflect the extension change in the nascent chain. At a constant force of 3.5 pN (blue segments, top panel), the 316_{RNC} molecule does not exhibit cooperative extension changes (bottom panel) that would indicate formation of a folding intermediate. The force is periodically jumped to 30 pN (red segments, top panel) to confirm that the nascent chain has remained in the unfolded state. The absence of folding and unfolding transitions at low and high force, respectively, indicates that the nascent chain remains unfolded at both forces. Figure 6C shows the extension of a low-force segment in greater detail.

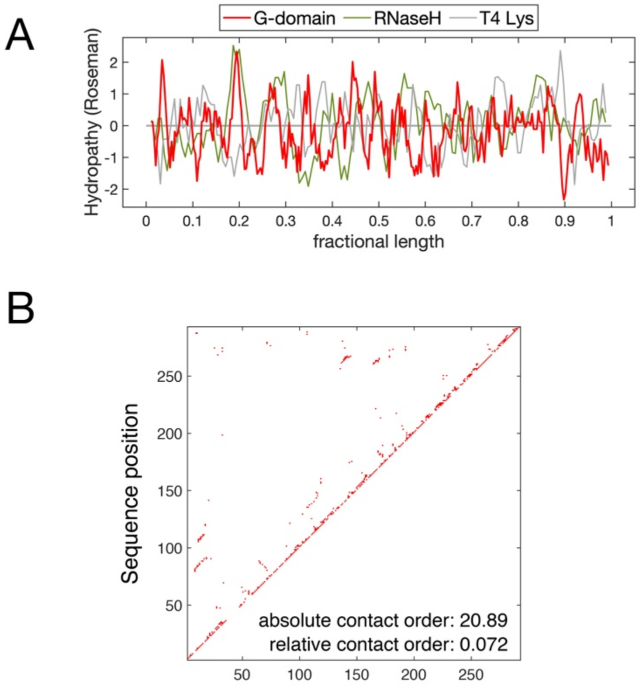

Supplementary Figure 5. Hydrophobicity and contact order of the G-domain. **A.** Hydrophobicity for the EF-G G-domain (red), calculated using the Roseman scale [4], averaged with a window size of 5 amino acids. Fractional length is the ratio of sequence position and total sequence length. The hydrophobicity profiles for ribonuclease H (olive) and T4 lysozyme (grey) are plotted for comparison, showing similar overall hydrophobicity. **B.** Contact map of the EF-G G-domain. Red squares indicate positions where residue pairs are in contact (atom distances within 6 Å). The absolute and relative contact order [5] are indicated in the plot.

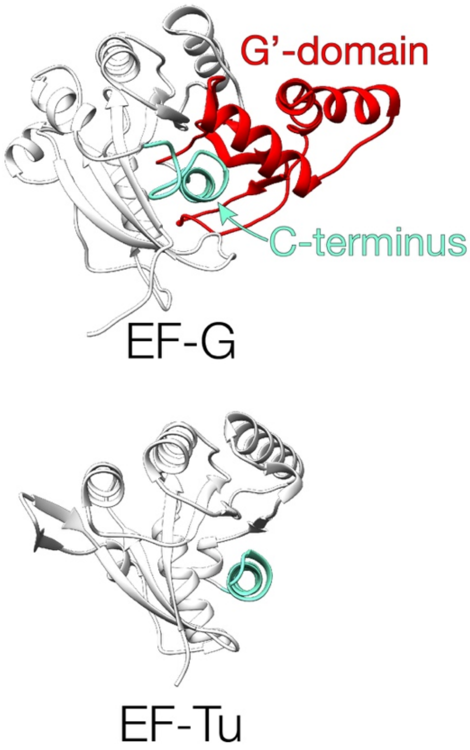

Supplementary Figure 6. Examples of G-domain structures. Cartoon representations of the G-domains from 4 elongation factors (EF-G, pdb: 4v9p; RF3, pdb: 2h5e; EF-Tu, pdb: 6eze; LepA: pdb: 3cb4). The C-terminal regions (aquamarine) in EF-G and RF3 are mostly buried due to the presence of the G' insertion (red) in these proteins. The same region is more surface accessible in EF-Tu and LepA, which lack the insertion. Cartoons were generated using Chimera [6].

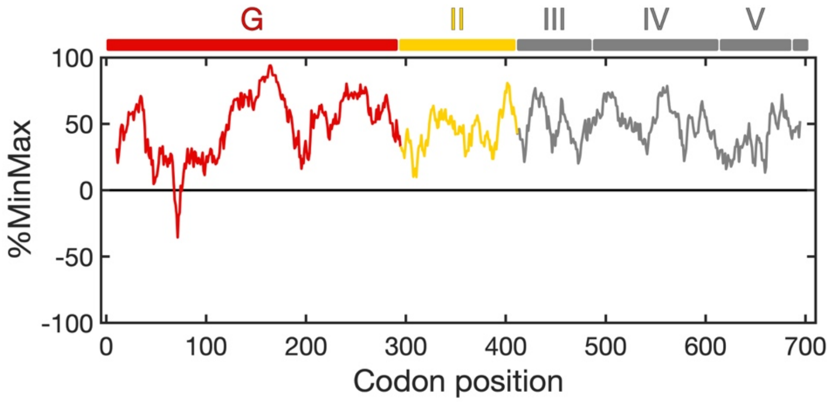

Supplementary Figure 7. Codon usage of the EF-G coding sequence. Codon usage over the complete EF-G coding sequence was calculated using a web interface (www.codons.org) based on the %MinMax algorithm [7] with a window size of 18. Domain boundaries in the EF-G protein are indicated on top. Clusters of rare codons are absent, except for a short region around codon position 70.

Supplementary Tables

Name	Sequence
gBlock	ATGGTTTTTACGCTGGAGGACTTCGTCGGTGACTGGCGTCAGACAGCGGGCTATAATCTTGATCAAG<br>TTTTAGAGCAAGGGGGCGTCTCCAGCCTTTTTCAAAACTTAGGCGTATCCGTCACGCCCATCCAACG<br>TATTGTATTGAGCGGGGAGAACGGATTAAAAATCGACATCCATGTCATCATTCCGTACGAAGGGCTTT<br>CCGGGGACCAGATGGGCCAAATCGAAAAGATTTTTAAGGTAGTTTATCCCGTTGACGATCACCACTTC<br>AAAGTGATTTTACACTACGGGACGCTTGTGATTGACGGAGTTACACCTAACATGATCGACTACTTTGG<br>ACGTCCCTACGAAGGTATTGCAGTATTTGATGGCAAAAAGATCACAGTGACTGGTACTTTGTGGAACG<br>GGAATAAGATTATTGATGAACGTTTGATTAACCCAGACGGTAGCCTTTTGTTTCGCGTGACCATCAATG<br>GTGTAACTGGTTGGCGCTTGTGCGAGCGCATCCTTGCGTAA
bb-rev	accgccaccactagtTTCGTGCCATTCGATTTTC
bb-fwd	TAAAGCTTGATCCGGCTGC
G-II_fwd	agaaaatcgaatggcacgaaactagtggtggcggtGCTCGTACAACACCCATC
G-II_rev	gacgggcgtgctgaaGATTTTGAACGCCAGTGC
SecM-nanoluc_rev	gttagcagccggatcaagctttaCGCAAGGATGCGCTCGCA
SecM-nanoluc-30_fwd	gttcaaaatcttcagcacgcccgtctggataagccaggcgcaaggcatccgtgctggccctGTTTTTACGCTGGAGGACTTCG<br>TCG

Supplementary Table 1: Synthetic DNA fragments used to construct plasmid pWP3. The gBlock contains the coding sequence for NanoLuc. The oligonucleotides below were used as primers to amplify DNA fragments by PCR that were then assembled into plasmid pWP3 by Gibson Assembly.

Name	Sequence
WP3-bb-fwd	CGCACTAGTTTCGTGCCATTCGATTTTCTGA
WP3-bb-rev	TTCAGCACGCCCGTCTGG
WP3-EFG-uni-fwd	CGCGCTAGCGGTGGCGGTGCTCGTACAACACCCATCG
WP3-EFG-44	GTCATGAACTTCACCGATTTTATGGTTTACACC
WP3-EFG-48	GGTTGCAGCGCCGTCATG
WP3-EFG-52	CATCCAGTCCATGGTTGCAGC
WP3-EFG-56	CTGCTCCTGCTCCATCCAGTC
WP3-EFG-60	AATACCACGTTCCTGCTCCTGC
WP3-EFG-64	GGAAGTGATGGTAATACCACGTTCCTG
WP3-EFG-68	AGTAGTCGCAGCGGAAGTGATG
WP3-EFG-72	AGACCAGAATGCAGTAGTCGCAG
WP3-EFG-76	CTTAGCCATACCAGACCAGAATGCAG
WP3-EFG-80	CGGCTCATACTGCTTAGCCATACC
WP3-EFG-84	GTTGATGCGATGCGGCTCATAC
WP3-EFG-88	GGTGTCGATGATGTTGATGCGATGC
WP3-EFG-92	AACGTGCCCCGGGGTG
WP3-EFG-96	GATTGTGAAGTCAACGTGCCCC
WP3-EFG-100	ACGTTCTACTTCGATTGTGAAGTCAACG
WP3-EFG-104	AACACGCATGGAACGTTCTACTTCG
WP3-EFG-108	CGCACCATCGAGAACACGC
WP3-EFG-112	GTAAACCATTACCGCACCATCGAG
WP3-EFG-116	ACCAACTGCGCAGTAAACCATTAC
WP3-EFG-120	CGGCTGAACACCACCAACTG
WP3-EFG-124	GGTTTCAGACTGCGGCTGAAC
WP3-EFG-128	CTGACGCCATACGGTTTCAGAC
WP3-EFG-132	ATATTTGTTTGCCTGACGCCATACG
WP3-EFG-136	GCGCGGAACTTTATATTTGTTTGCC
WP3-EFG-140	AACGAACGCAATGCGCGG
WP3-EFG-144	GTCCATTTTGTTAACGAACGCAATGCG
WP3-EFG-148	CGCACCCATGCGGTCC
WP3-EFG-152	TTTCAGGAAGTTCGCACCCATGC
WP3-EFG-156	CTGGTTAACAACTTTCAGGAAGTTCGC
WP3-EFG-160	ACGGGTTTTGATCTGGTTAACAACTTTC
WP3-EFG-164	GTTCGCGCCCAGACGG
WP3-EFG-168	CAGCGGAACCGGGTTCG
WP3-EFG-172	AATCGCCAGCTGCAGCG
WP3-EFG-176	TTCTTCAGCACCAATCGCCAGC
WP3-EFG-180	ACCGGTGAAATGTTCTTCAGCAC
WP3-EFG-184	CAGGTCAACAACACCGGTGAAATG
WP3-EFG-188	TTTCATTTTCACCAGGTCAACAACACC
WP3-EFG-192	CCAGTTGATAGCTTTCATTTTCACCAGG
WP3-EFG-196	GTCAGCGTCGTTCCAGTTGATAGC
WP3-EFG-200	GGTTACGCCCTGGTCAGCG
WP3-EFG-204	TTCGTATTCGAAGGTTACGCCCTG
WP3-EFG-208	TGCCGGGATATCTTCGTATTCGAAG
WP3-EFG-212	TTCAACCATGTCTGCCGGGATATC
WP3-EFG-216	TTCGTTAGCCAGTTCAACCATGTCTG
WP3-EFG-220	GTTCTGGTGCCATTCGTTAGCC
WP3-EFG-224	GGATTCGATCAGGTTCTGGTGCC
WP3-EFG-228	AGCTTCAGCTGCGGATTCG

Name	Sequence
WP3-EFG-232	CAGCTCTTCAGAAGCTTCAGCTG
WP3-EFG-236	GTATTTTTCCATCAGCTCTTCAGAAGCTTCAG
WP3-EFG-240	TTCACCACCCAGGTATTTTTCCATCAG
WP3-EFG-244	TTCAGTCAGTTCTTCACCACCCAG
WP3-EFG-248	TTTGATTTCTGCTTCAGTCAGTTCTTCACC
WP3-EFG-252	ACGCAGAGCACCTTTGATTTCTG
WP3-EFG-256	CAGAACGCGCTGACGCAG
WP3-EFG-260	GATTTCGTTGTTCAGAACGCGC
WP3-EFG-264	GGTTACCAGGATGATTTCGTTGTTCAGAAC
WP3-EFG-268	CGCAGAACCACAGGTTACCAGG
WP3-EFG-272	TTTGTTCTTGAACGCAGAACCACAGG
WP3-EFG-276	CGCCTGAACACCTTTGTTCTTGAAC
WP3-EFG-280	CGCATCCAGCATCGCCTG
WP3-EFG-284	GTAATCAATTACCGCATCCAGCATCGC
WP3-EFG-288	CGGGGATGGCAGGTAATCAATTACC
WP3-EFG-292	AGGTACGTCAACCGGGGATG
WP3-EFG-296	ACCGTTGATCGCAGGTACG
WP3-EFG-300	GTCGTCCAGGATACCGTTGATCG
WP3-EFG-304	AGTGTCTTTACCGTCGTCCAGG
WP3-EFG-308	ACGTTCAGCCGGAGTGTCTTTAC
WP3-EFG-312	ATCACTTGCGTGACGTTCAGC
WP3-EFG-316	GAACGGCTCGTCATCACTTGC
WP3-EFG-320	CGCCAGTGCAGAGAACGG
WP3-EFG-324	AGCGATTTTGAACGCCAGTGC
WP3-EFG-328	AAACGGGTCGGTAGCGATTTTG
WP3-EFG-332	CAGGTTACCAACAAACGGGTCG
WP3-EFG-336	ACGGAAGAAGGTCAGGTTACCAAC
WP3-EFG-340	ACCGGAGTAAACACGGAAGAAGG
WP3-EFG-344	AGAGTTAACCACACCGGAGTAAACAC
WP3-EFG-348	TACGGTATCACCAGAGTTAACCACACC
WP3-EFG-352	CACGGAGTTCAGTACGGTATCACC
WP3-EFG-356	ACGTGCAGCTTTCACGGAG
WP3-EFG-360	ACCGAAACGCTCACGTGC
WP3-EFG-364	CTGAACGATACGACCGAAACGC
WP3-EFG-368	GTTAGCGTGCATCTGAACGATACGAC
WP3-EFG-372	CTCTTCACGTTTGTTAGCGTGCATC
WP3-EFG-376	AACTTCTTTGATCTCTTCACGTTTGTTAGCG
WP3-EFG-380	GTCGCCCGCGCGAAC
WP3-EFG-384	AGCAGCAGCGATGTCGC
WP3-EFG-388	TTTCAGACCGATAGCAGCAGCG
WP3-EFG-392	AGTGGTTACGTCTTTCAGACCGATAG
WP3-EFG-396	CAGGGTGTCACCAGTGGTTACG
WP3-EFG-400	ATCCGGGTCACACAGGGTG
WP3-EFG-404	AATGATCGGCGCATCCGG
WP3-EFG-408	CATACGTTCCAGAATGATCGGCG
WP3-EFG-412	CTCAGGGAATTCCATACGTTCCAGAATG
WP3-EFG-416	GGAGATTACCGGCTCAGGGAATTC
WP3-EFG-420	TTCAACTGCGATGGAGATTACCGG
WP3-EFG-424	TTTGGTTTTCGGTTCAACTGCGATG

Supplementary Table 2. Primers used for constructing the EF-G truncation library. Primers WP3-bb-fw and WP3-bb-rv were used to amplify the backbone of plasmid pWP3. Primer WP3-EFG-uni-fwd was used in combination with one of the other primers listed here to generate PCR products encoding EF-G fragments of the specified sizes that were subsequently ligated with the backbone PCR product.

Name	Sequence
WP3 fw T7	GTCGGCGATATAGGCGCCAG
WP3-EFG-44	GTCATGAACTTCACCGATTTTATGGTTTACACC
WP3-EFG-252	ACGCAGAGCACCTTTGATTTCTG
WP3-EFG-316	GAACGGCTCGTCATCACTTGC
WP3-EFG-328	AAACGGGTCGGTAGCGATTTTG

Supplementary Table 3. Primers used for generating templates for *in vitro* transcription. Primer WP3 fwT7 was used in combination with one of the other primers listed here to generate PCR products that served as templates for *in vitro* transcription. The products of *in vitro* transcription then served as templates for translation to generate stalled RNCs used in optical tweezers experiments.

Supplementary References

1. Hall, M.P. et al. Engineered luciferase reporter from a deep sea shrimp utilizing a novel imidazopyrazinone substrate. *ACS Chem Biol* **7**, 1848-57 (2012).
2. Liu, K., Maciuba, K. & Kaiser, C.M. The Ribosome Cooperates with a Chaperone to Guide Multi-domain Protein Folding. *Mol Cell* **74**, 310-319 e7 (2019).
3. Liu, K., Rehfus, J.E., Mattson, E. & Kaiser, C.M. The ribosome destabilizes native and non-native structures in a nascent multidomain protein. *Protein Sci* **26**, 1439-1451 (2017).
4. Roseman, M.A. Hydrophilicity of polar amino acid side-chains is markedly reduced by flanking peptide bonds. *J Mol Biol* **200**, 513-22 (1988).
5. Plaxco, K.W., Simons, K.T. & Baker, D. Contact order, transition state placement and the refolding rates of single domain proteins. *J Mol Biol* **277**, 985-94 (1998).
6. Pettersen, E.F. et al. UCSF Chimera--a visualization system for exploratory research and analysis. *J Comput Chem* **25**, 1605-12 (2004).
7. Clarke, T.F.t. & Clark, P.L. Rare codons cluster. *PLoS One* **3**, e3412 (2008).
